# Supplementary material for: Immunogenicity to COVID-19 mRNA vaccine third dose in people living with HIV
Source: Nat Commun. 2022 Aug 22;13:4922. doi: 10.1038/s41467-022-32263-7 (PMC9395398; doi:10.1038/s41467-022-32263-7)
Supplement: Supplementary file 1 — Supplementary Information [file 41467_2022_32263_MOESM1_ESM.pdf]

Immunogenicity to COVID-19 mRNA vaccine third dose in people living with HIV

Vergori Alessandra[1*§], Cozzi Lepri Alessandro[2§], Cicalini Stefania[1], Matusali Giulia[3], Bordoni Veronica[4], Lanini Simone[1], Meschi Silvia[3], Iannazzo Roberta[1], Mazzotta Valentina[1], Colavita Francesca[3], Mastrorosa Ilaria[1], Cimini Eleonora[4], Mariotti Davide[4], De Pascale Lydia[1], Marani Alessandra[5], Gallì Paola[5], Garbuglia AnnaRosa[3], Castilletti Concetta[3], Puro Vincenzo[6], Agrati Chiara[4], Girardi Enrico[7], Vaia Francesco[5], Antinori Andrea[1] on behalf of the HIV-VAC study group

§ Both authors contributed equally

[1] HIV/AIDS Unit, National Institute for Infectious Diseases Lazzaro Spallanzani IRCCS, Rome, Italy

[2]Centre for Clinical Research, Epidemiology, Modelling and Evaluation (CREME), Institute for Global Health, UCL, London, UK

[3]Laboratory of Virology, National Institute for Infectious Diseases Lazzaro Spallanzani IRCCS, Rome, Italy

[4]Laboratory of Cellular Immunology and Clinical Pharmacology, National Institute for Infectious Diseases Lazzaro Spallanzani IRCCS, Roma, Italy

[5] Health Direction, National Institute for Infectious Diseases Lazzaro Spallanzani IRCCS, Roma, Italy

[6] Risk management Unit, National Institute for Infectious Diseases Lazzaro Spallanzani IRCCS, Roma, Italy

[7]Scientific Direction, National Institute for Infectious Diseases Lazzaro Spallanzani IRCCS, Roma, Italy

Corresponding author

Alessandra Vergori, MD

HIV/AIDS Unit

National Institute for Infectious Diseases Lazzaro Spallanzani IRCCS

Via Portuense 292, 00149 Roma, Italy

Phone: +39 06 55170546

Fax: +39 06 55170477

e-mail: alessandra.vergori@inmi.it

Supplementary Material

Supplementary figure 1. Model assumptions for CD4 a T0 as exposure.

This model assumption is described by means of a direct acyclic graph (DAG), built using DAGitty vers. 2.3 released 2015-08-19, available at http://www.daggity.net/ and the following variables have been identified as potential confounders for the association between CD4 count and immunogenic responses: age, time from HIV diagnosis, CD4 nadir, HIV-RNA at the time of third dose, days from the date of 2nd dose, vaccine sequence and concomitant cancer.

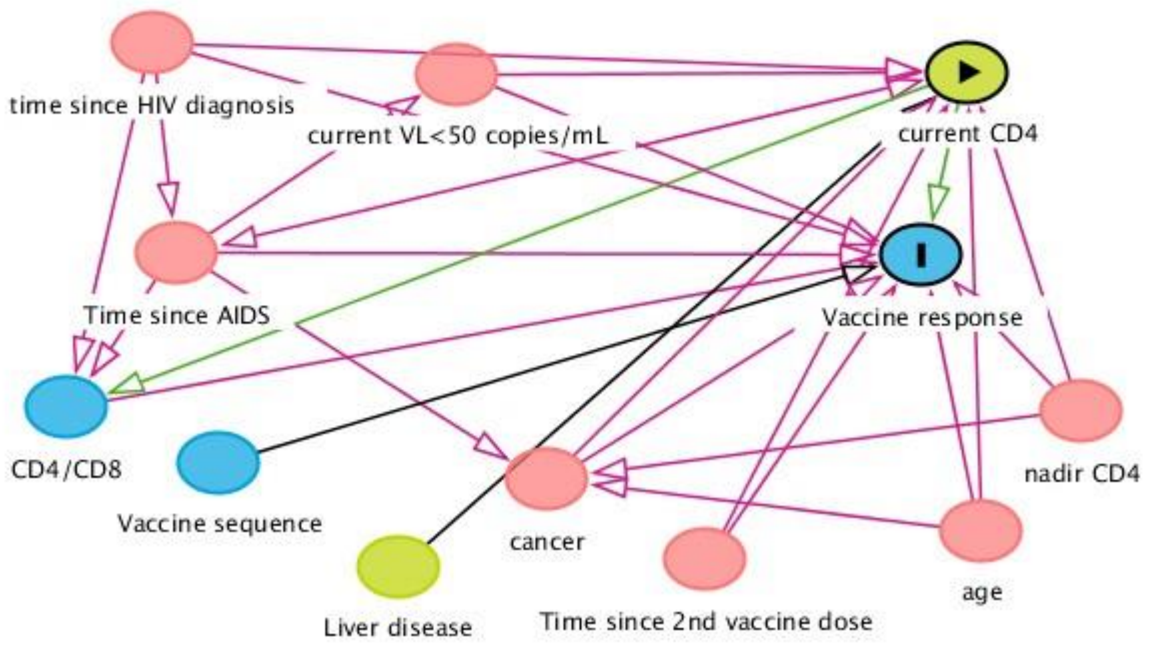

Supplementary figure 2 A-C. Mean log_2 change of anti-RBD IgG from 1 month response after the 2nd dose (T_{-1}) and after the 3rd dose (T1) (Fisher test p= 0.003) in PCDR(A), ICDR (B), HCDR(C).

Mean Log_2 values of anti-RBD IgG did not differ significantly between PCDR and ICDR, between PDCR and HCDR; conversely, a significant difference was found for ICDR vs HCDR. Source data are provided as a Source Data.

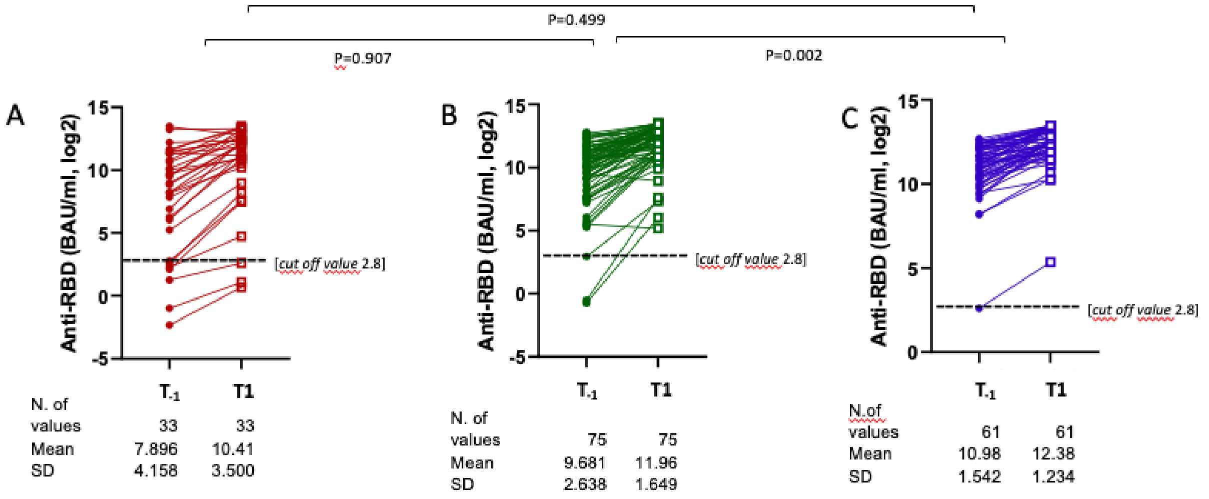

Supplementary figure 3 A-C. Mean log_2 change of nAbs from 1 month response after the 2nd dose (T_{-1}) and after the 3rd dose (T1) (fisher test p=0.006) in PCDR (A), ICDR (B), HCDR(C) (p-values above the figure panels are Bonferroni-corrected values).

Mean Log_2 values of neutralizing antibodies (MNA_{90}) did not differ significantly among the three group of participants. Source data are provided as a Source Data file.

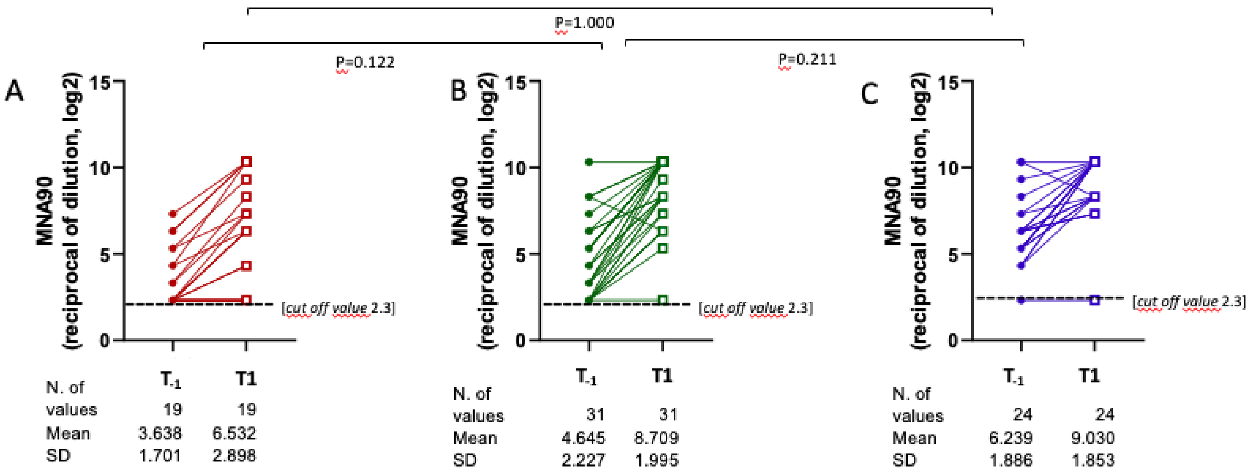

Supplementary figure 4 A-C. Mean log2 change of IFN-γ from 1 month response after the 2nd dose (T_{-1}) and after the 3rd dose (T1) (fisher test p=0.3) in PCDR(A), ICDR (B), HCDR(C). Mean Log_2 values of IFN-γ did not differ significantly among the three group of participants. Source data are provided as a Source Data file.

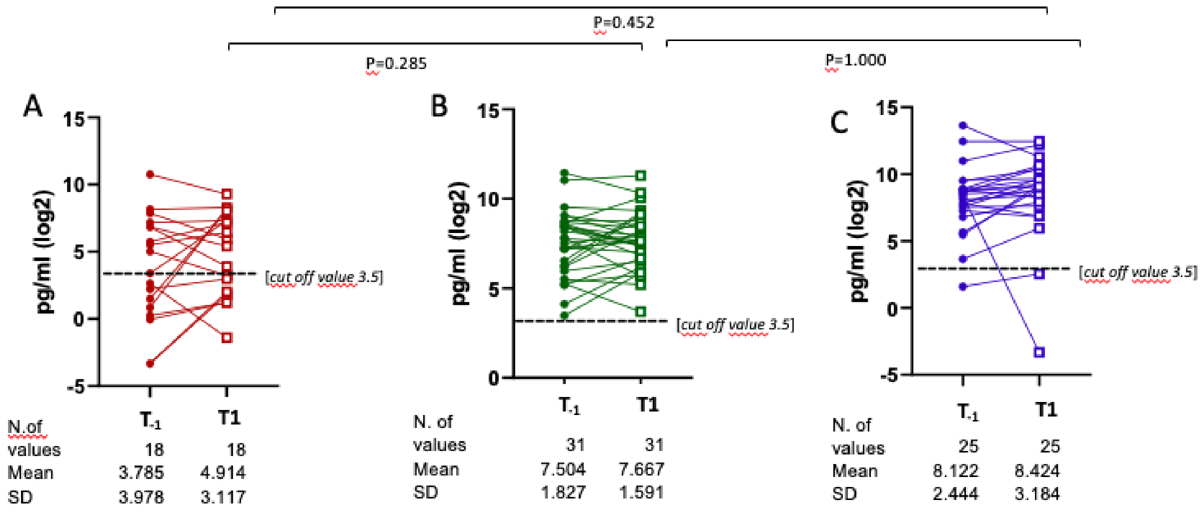

Supplementary figure 5A-C. Mean Δ changes of anti-RBD IgG (A), nAbs (B) and IFN-γ (C) by CD4 count groups.
Legend: Red circles represent mean Δ changes of anti RBD IgG, MNA900 and IFN-γ in PCDR; green circles represent mean Δ changes of anti RBD IgG, MNA90 and IFN-γ in ICDR and blue circles represent mean Δ changes of anti RBD IgG, MNA90 and IFN-γ in HCDR. Source data are provided as a Source Data file.

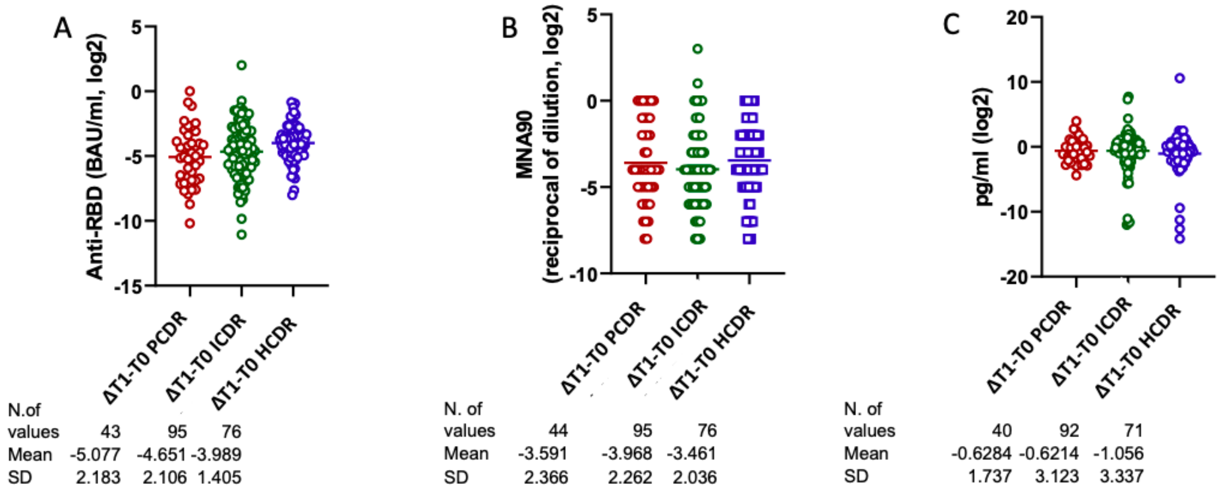

Supplementary figure 6 A-C Mean log2 change of anti RBD IgG (A), nAbs (B) and IFN-γ (C) at T1 according to vaccine combination; Unpaired t-test, 2-sided with Bonferroni adjusted p-values for multiple comparisons. Legend: Red circles represent values of anti RBD IgG (A), nAbs (B) and IFN-γ (C) before and after third dose vaccine with the combination of mRNA1273 for all the three doses, while red cross the mean Δ change between T1 and T0; Green circles represent values of anti of anti RBD IgG (A), nAbs (B) and IFN-γ (C) before and after third dose vaccine with the combination of mRNA1273 as primary cycle and the third dose with BNT162b2, while green cross the mean Δ change between T1 and T0; Blue circles represent values of of anti RBD IgG (A), nAbs (B) and IFN-γ (C) before and after third dose vaccine with the combination of BNT162b2as primary cycle and the third dose with mRNA1273, while blue cross the mean Δ change between T1 and T0; Violet circles represent values of of anti RBD IgG (A), nAbs (B) and IFN-γ (C)before and after third dose vaccine with the combination of BNT162b2 for all the three doses, while violet cross the mean Δ change between T1 and T0. Source data are provided as a Source Data file.

A

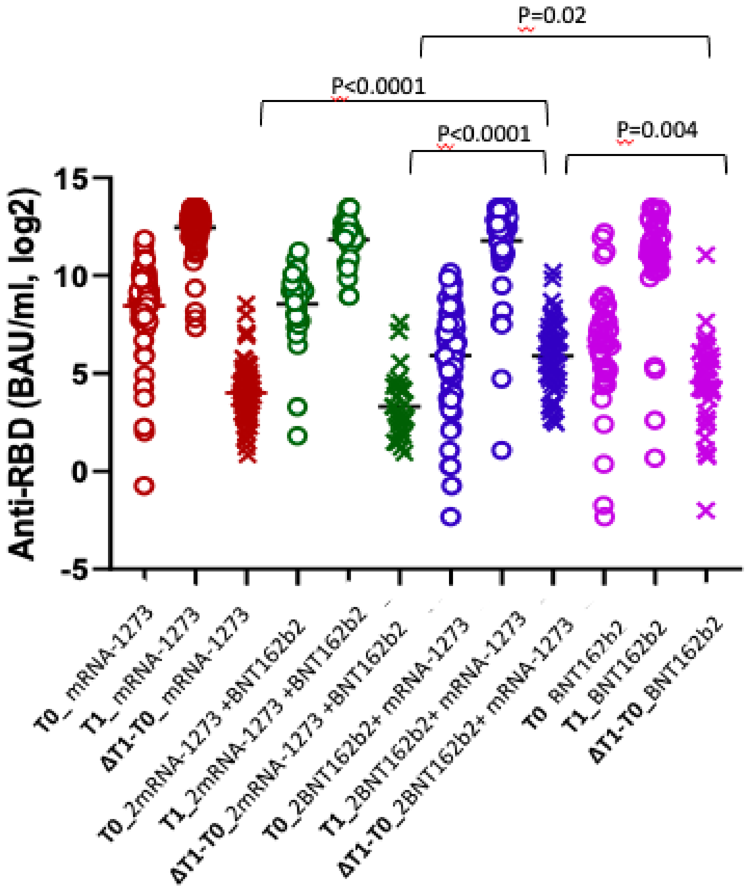

N. of values	80	80	80	31	31	31	57	57	57	44	44	44
Mean	8.4	12.4	4.0	8.5	11.8	3.3	5.9	11.8	5.9	6.3	10.9	4.5
SD	2.0	1.2	1.4	1.9	1.1	1.6	2.7	2.3	1.7	2.9	2.6	2.1

B

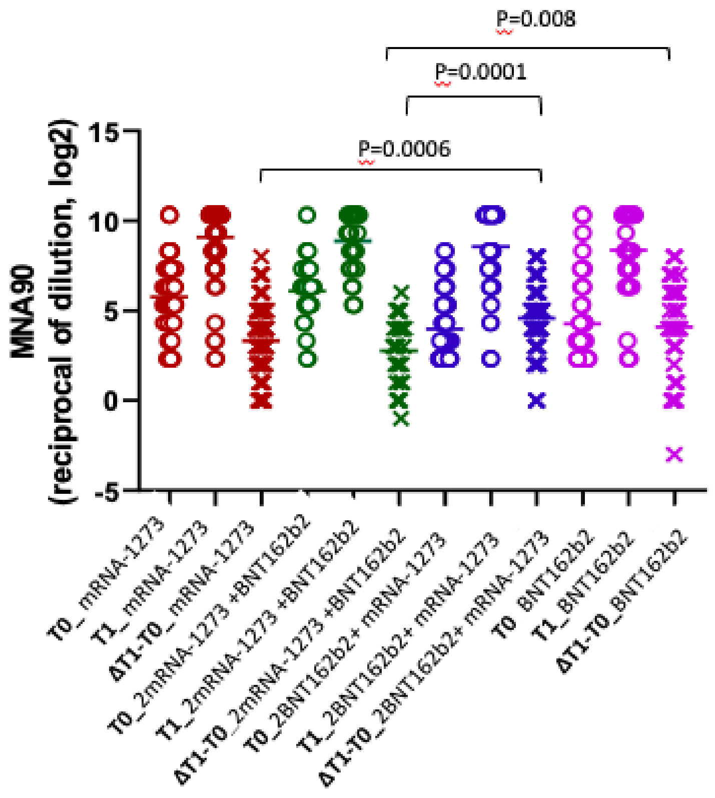

N. of values	81	81	81	31	31	31	57	57	57	44	44	44
Mean	5.7	9.0	3.3	6.1	8.8	2.7	3.9	8.5	4.6	4.2	8.3	4.1
SD	1.9	1.9	1.9	1.7	1.5	1.8	1.8	2.3	2.0	2.2	2.3	2.5

C

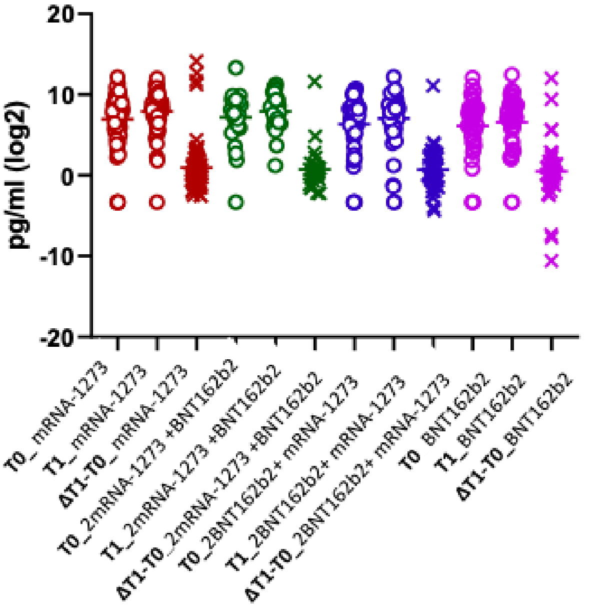

N. of values	77	77	77	29	29	29	54	54	54	41	41	41
Mean	6.9	7.9	0.9	7.1	7.9	0.7	6.3	7.0	0.7	6.1	6.5	0.4
SD	3.5	2.2	3.1	3.1	2.2	2.5	3.5	3.2	2.3	3.6	3.3	3.8

Supplementary figure 7. Overall self-reported solicited local and systemic reactions in days 0–7 after vaccination with third additional dose by vaccine combination group.

Legend: mRNA-1273 (for all the three doses); 2mRNA-1273+ BNT162b2 (2 doses of mRNA-1273 + BNT162b2 for 3 AD); **2**BNT162b2 + mRNA-1273 (2 doses of **2**BNT162b2 + mRNA-1273 for 3AD); BNT162b2 (for all the 3 doses)

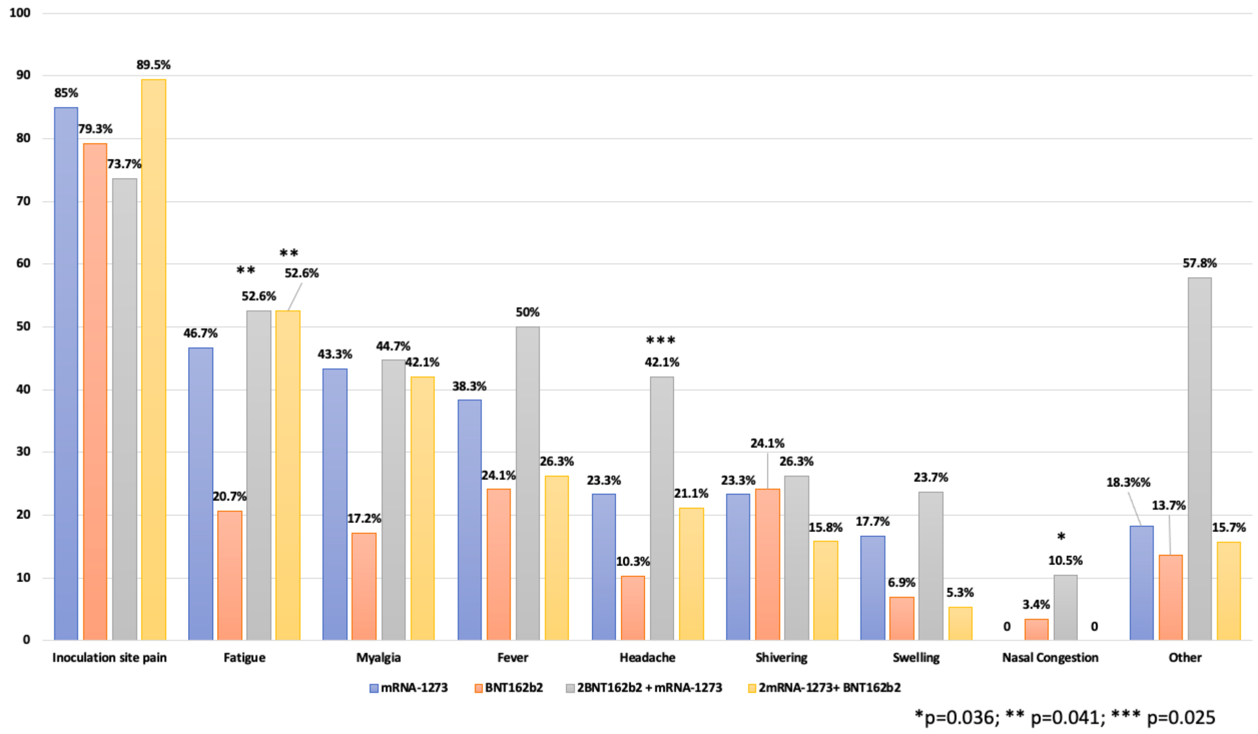

Supplementary Table 1. Transition in CD4 count from baseline to T0 (time of third dose)

**Baseline CD4 (cell/mm^3)**	**T0 group**			
	**PCDR**	**ICDR**	**HCDR**	**Total**
**0-200**	42 (74%)	15 (26%)	0 (15%)	57 (100%)
**201-500**	2 (2%)	74 (81%)	15 (16%)	91 (100%)
**500+**	0 (0%)	7 (10%)	61 (90%)	68 (100%)
**Total**	44	96	76	216
