# Supplementary material for: Immunogenicity to COVID-19 mRNA vaccine third dose in people living with HIV
Source: Nat Commun. 2022 Aug 22;13:4922. doi: 10.1038/s41467-022-32263-7 (PMC9395398; doi:10.1038/s41467-022-32263-7)
Supplement: Supplementary file 3 — Reporting Summary [file 41467_2022_32263_MOESM3_ESM.pdf]

nature portfolio

Corresponding author(s): Alessandra Vergori

Last updated by author(s): Jul 2, 2022

Reporting Summary

Nature Portfolio wishes to improve the reproducibility of the work that we publish. This form provides structure for consistency and transparency in reporting. For further information on Nature Portfolio policies, see our Editorial Policies and the Editorial Policy Checklist.

Statistics

For all statistical analyses, confirm that the following items are present in the figure legend, table legend, main text, or Methods section.

n/a	Confirmed	
☐	☒	The exact sample size (*n*) for each experimental group/condition, given as a discrete number and unit of measurement
☐	☒	A statement on whether measurements were taken from distinct samples or whether the same sample was measured repeatedly
☐	☒	The statistical test(s) used AND whether they are one- or two-sided<br>*Only common tests should be described solely by name; describe more complex techniques in the Methods section.*
☐	☒	A description of all covariates tested
☐	☒	A description of any assumptions or corrections, such as tests of normality and adjustment for multiple comparisons
☐	☒	A full description of the statistical parameters including central tendency (e.g. means) or other basic estimates (e.g. regression coefficient) AND variation (e.g. standard deviation) or associated estimates of uncertainty (e.g. confidence intervals)
☐	☒	For null hypothesis testing, the test statistic (e.g. *F*, *t*, *r*) with confidence intervals, effect sizes, degrees of freedom and *P* value noted<br>*Give P values as exact values whenever suitable.*
☒	☐	For Bayesian analysis, information on the choice of priors and Markov chain Monte Carlo settings
☒	☐	For hierarchical and complex designs, identification of the appropriate level for tests and full reporting of outcomes
☒	☐	Estimates of effect sizes (e.g. Cohen's *d*, Pearson's *r*), indicating how they were calculated

Our web collection on statistics for biologists contains articles on many of the points above.

Software and code

Policy information about availability of computer code

Data collection	No software has been used for data collection
Data analysis	SAS Version 9.4 SAS Institute Inc., Cary, NC, USA; GraphPad Prism 9.0 (GraphPad Software, Inc., San Diego, CA); DAGitty vers. 2.3 released 2015-08-19, available at http://www.daggity.net/

For manuscripts utilizing custom algorithms or software that are central to the research but not yet described in published literature, software must be made available to editors and reviewers. We strongly encourage code deposition in a community repository (e.g. GitHub). See the Nature Portfolio guidelines for submitting code & software for further information.

Data

Policy information about availability of data

All manuscripts must include a data availability statement. This statement should provide the following information, where applicable:

- Accession codes, unique identifiers, or web links for publicly available datasets
- A description of any restrictions on data availability
- For clinical datasets or third party data, please ensure that the statement adheres to our policy

The data repository is owned by INMI L.Spallanzani and is available upon reasonable request. We are extremely concerned about confidentiality – since these patients may be identified by combinations of person-specific characteristics within the database, and the database includes sensitive data such as HIV status, as well as data on intravenous drug use as well as sexual preferences. Moreover, this is an ongoing study, funded by the Italian Ministry of Health and supported by the Italian Drug Agency (AIFA), and the database is currently being analysed for several other projects. We are concerned that public access to our dataset would compromise our ability to fulfill the milestones of the project.
For all these reasons we have decided to share the database upon reasonable request.

Field-specific reporting

Please select the one below that is the best fit for your research. If you are not sure, read the appropriate sections before making your selection.

- [x] Life sciences
- [ ] Behavioural & social sciences
- [ ] Ecological, evolutionary & environmental sciences

For a reference copy of the document with all sections, see nature.com/documents/nr-reporting-summary-flat.pdf

Life sciences study design

All studies must disclose on these points even when the disclosure is negative.

Sample size	No sample size calculations were performed. Participants in this analysis are all PLWH who underwent the additional booster dose of the vaccine. It is a subset of those who, following written informed consent, had been enrolled in an observational cohort study to evaluate the outcomes of SARS-CoV-2 vaccination (the HIV-VAC study). Most of the 95% CI in the analysis excluded the value under the null hypothesis which is indirect evidence that the analysis was adequately powered.
Data exclusions	Individuals with a SARS-CoV-2 infection diagnosis, defined by a RT-PCR positive to the molecular test on the nasopharyngeal swab, or positivity to anti-N IgG at T0, were excluded for the present analysis. The rationale was to exclude PLWH in whom SARS CoV2 infection might have altered the magnitude of the vaccine response
Replication	All serology results can be replicated. Both anti-S and anti-N IgG were detected using commercial tests. Neutralisation test was performed following standard protocols and using the WHO/NIBSC standard to normalize and obtain reproducing results.For this analysis tests have been performed once, independently.
Randomization	This is an observational study, participants have not been randomized
Blinding	Laboratory personel were blind to whether the sample belonged to the PCDR, ICDR, HCDR or HCW group

Reporting for specific materials, systems and methods

We require information from authors about some types of materials, experimental systems and methods used in many studies. Here, indicate whether each material, system or method listed is relevant to your study. If you are not sure if a list item applies to your research, read the appropriate section before selecting a response.

Materials & experimental systems

n/a	Involved in the study	
☐	☒	Antibodies
☐	☒	Eukaryotic cell lines
☒	☐	Palaeontology and archaeology
☒	☐	Animals and other organisms
☐	☒	Human research participants
☐	☒	Clinical data
☒	☐	Dual use research of concern

Methods

n/a	Involved in the study	
☒	☐	ChIP-seq
☒	☐	Flow cytometry
☒	☐	MRI-based neuroimaging

Antibodies

Antibodies used	WHO International Standard First WHO International Standard for anti-SARS-CoV-2 immunoglobulin (human) NIBSC code: 20/136 (datasheet available at: https://www.nibsc.org/documents/ifu/20-136.pdf, last accessed :March, 15, 2022)
Validation	The international standard used is a poolof human sera from convalescent COVID19 patients. We did not use any primary antibody.

Eukaryotic cell lines

Policy information about cell lines

Cell line source(s)	We used Vero E6 cell obtained from ATCC, Manassas, Virginia, United States; catalog n. CRL-1586™
Authentication	Vero E6 cells are authenticated by ATCC
Mycoplasma contamination	Cell line used was periodically tested for mycoplasma contamination testing negative by PCR assay

Commonly misidentified lines (See ICLAC register)	N/A

Human research participants

Policy information about studies involving human research participants

Population characteristics	A total of 216 PLWH were included in the analysis (PCDR=44; ICDR =96; HCDR =76). The median age was 54 years old (IQR 47, 59); all HIV patients were on ART at time of the third dose, 92.6% had HIV-RNA <50 copies/mL with a median time since HIV diagnosis of 7 years (3-12) and of 5 years (2-8) since AIDS, if diagnosed; 6.9 % with a diagnosis of cancer. A control group of HIV negative health care workers have been included in the analysis, was mainly composed by female subjects (73.5%) vs 18% in PLWH; p<0.0001], with a younger median age 44 years old (IQR 32-52) than PLWH (54 years old (47-59), the median time between the 3D and response measurement was 16 days (14-18), shorter than that observed in PLWH [33 days (30-35)], as well as, the interval of response measurement between T-1 and T0 was was significantly longer than PLWH [285 days (280, 291) vs 117 (103,126)].
Recruitment	PLWh attending our center for routine clinical care were offered SARS-CoV-2 vaccination and participation in this observational study. A subgroup of consecutively enrolled patients were included for this analysis, so a subset of PLWH who on September 10th, 2021, took part of the Nationwide Booster Vaccination Program in Italy, the National Institute for Infectious Diseases Lazzaro Spallanzani in Rome who started the boosting vaccination against SARS-CoV-2 in PLWH, as a third additional dose of a mRNA vaccine (a full single dose of BNT162b2 or mRNA-1273).
Ethics oversight	Ethical Committee of the Lazzaro Spallanzani Institute, as National Review Board for COVID-19 pandemic in Italy (approval number 423/2021)

Note that full information on the approval of the study protocol must also be provided in the manuscript.

Clinical data

Policy information about clinical studies

All manuscripts should comply with the ICMJEguidelines for publication of clinical research and a completedCONSORT checklist must be included with all submissions.

Clinical trial registration	AIFA approval: 66/OSS
Study protocol	Details of the study protocol have been described elsewhere (Antinori A, et al. Clin Infect Dis. 2022 Apr 2:ciac238).
Data collection	Humoral and neutralizing antibodies responses were retrospectively measured in blood samples which were stored at time of the third dose (T0) and approximately 15 days after the third dose (T1). T-cell response was measured on fresh blood collected at the same times. In addition, both humoral neutralizing antibodies and T-cell responses measured approximately 30 days after participants' second vaccine dose were also available for comparative analysis (time T-1). In addition, at day 7 after the third dose, participants were asked via a telephone interview about solicited adverse events which might have occurred over the period 0-7 days following the third dose.
Outcomes	The primary outcome was immunogenicity (humoral, neutralizing and cell-mediated responses) measured 15 days after receiving the third dose (T1), to investigate the role of CD4 count at the time of vaccination and comparison with a HIV negative control group
